# Peer Gynt’s last crossroad: the loneliness of an old adventurer

**DOI:** 10.1007/s41999-023-00805-x

**Published:** 2023-05-31

**Authors:** Marte Rognstad Mellingsæter, Marius Myrstad

**Affiliations:** 1 Department of Geriatric Medicine, Akershus University Hospital, Nordbyhagen, Norway; 2 Department of Internal Medicine, Bærum Hospital Vestre Viken Hospital Trust, Gjettum, Norway

**Keywords:** Art, Theatre, Frailty, Ageing

The Norwegian actor Toralv Maurstad died on November 4th 2022, at the age of 95. To many Norwegians, he represented an incarnation of Peer Gynt, the character in Henrik Ibsen’s famous play. Maurstad, a major cultural figure in Norway, played Peer Gynt in numerous productions throughout a period of almost 70 years, most recently at the age of 92.

The Norwegian playwright and poet Henrik Ibsen (1828–1906) is the second most performed playwright worldwide, after Shakespeare. His international breakthrough “A doll’s house”, published in 1879, is considered the start of modern, realistic drama and still contributes to social debate. “Peer Gynt”, a five-act play in verse, was written in 1867, during the romantic period. The original music for the play was written by the contemporaneous composer Edvard Grieg. Grieg was another famous representative of romanticism, a great period for many art disciplines in Norway. The music remains part of standard repertoire, and includes some of today’s most recognised classical pieces worldwide [1]. For Norwegians, “Peer Gynt” and its original music are inextricably linked to both the country’s beautiful natural environment, characterised by the steep mountains and deep fjords, and parts of the Norwegian identity, as self-made and rural.

“Peer Gynt” is a chronological story following Peer’s life from youth to old age. Peer is an unprincipled and irresponsible character, only true to his own wishes and needs. From village gossip, we know Peer’s father drank too much and died early, and that his mother drowned the shame in lies and fairy tales. Confronted with difficulties, Peer shies away from reality and makes up stories. On his adventurous way, he faces trolls and monsters, such as “the Bøyg”: in Norwegian folklore this represents something impossible to overcome. The Bøyg’s saying *“Go roundabout, Peer! *[2]*”* becomes a motto to Peer. Confronted with the fear of responsibility and upcoming trouble, he leaves Solveig, the innocent, pure, Christian girl who loves him. *“Be patient, my girl; be my way long or short – you must wait.”*

In Maurstad’s last performance as Peer Gynt, in a production performed at ‘Det Norske Teater’ in Oslo in 2018–19, five different actors play the character of Peer as he increases in age [3]. Meanwhile, Maurstad plays the older Peer who is present at the stage throughout most of the play, alternately observing and commenting on the younger Peer’s ego.

**Fig. 1:**
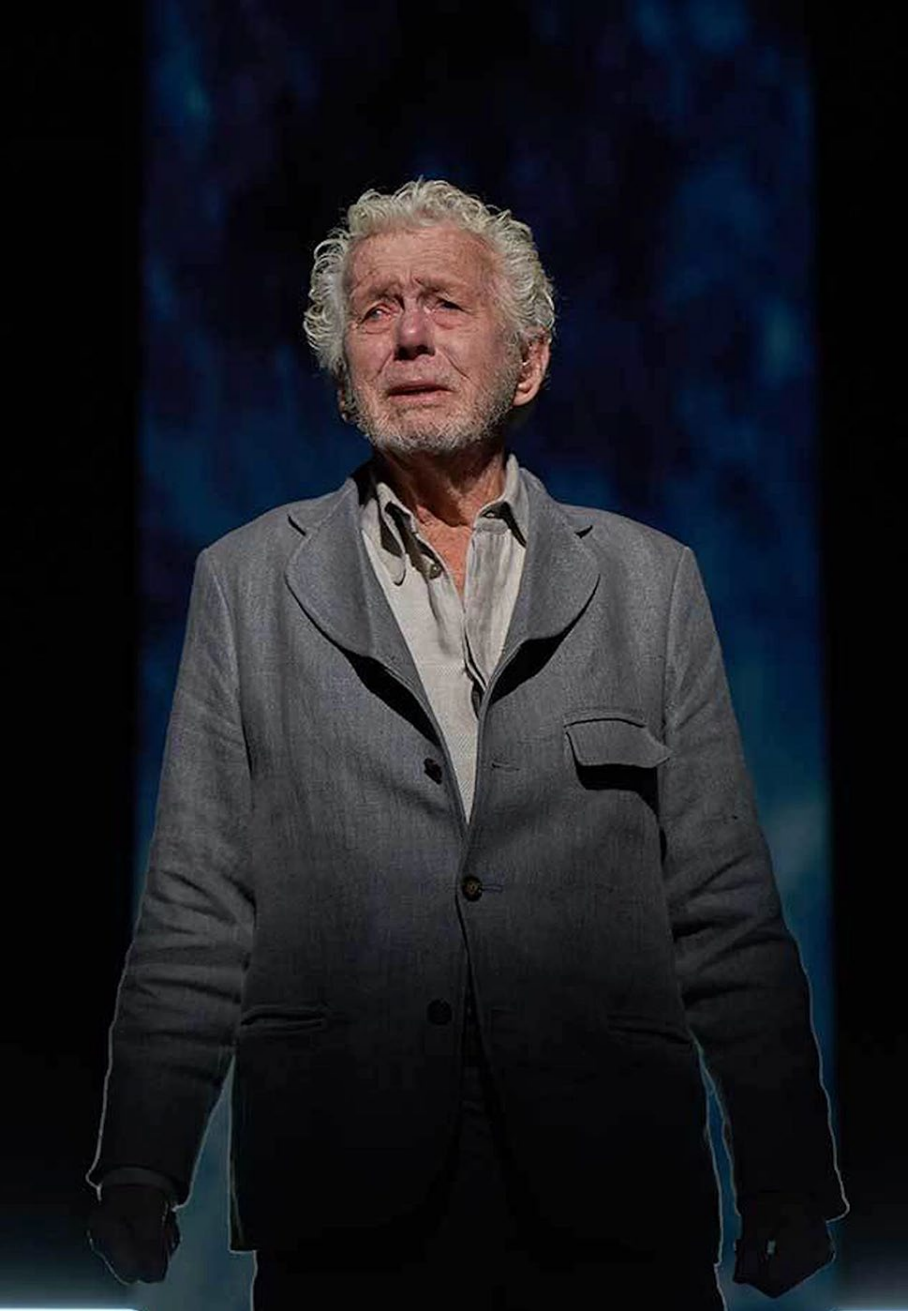
The Norwegian actor Toralv Maurstad played Peer Gynt in numerous productions throughout a period of almost 70 years, most recently at the age of 92. (Photo: Stig Håvard Dirdal)

Despite Maurstad’s remarkable endurance as an actor, we recognise signs of ageing and frailty. His posture and voice reveals lived life, and he moves with the pace of an elderly man, trying to be nothing but a nonagenarian. Wearing a benevolent grey suite, as if to emphasise that a costume is redundant in this matter. He adds outstanding credibility to the character and leaves us with the question if it could be his own, or anyone’s life and ego he so critically observes (Fig. 1).

The director Erik Ulfsby has not attempted to cover any weakness or frailty. Instead, he has embraced the age and constitution of the actor as a powerful means of action. Maurstad has a bottle of water within reach and sips from it during the long play. He might have a dry mouth, a common complaint at his age. At times, he seems to make a side step, suggesting his balance is not what it used to be. The rich text in verses is challenging to anyone, requiring cognitive capacities such as attention and deferred recall, but Maurstad controls it as his own. Any hesitation or slowness provides us with the moment we need to have glimpses of insight, to feel the insecurity, loss and sorrow of Peer. The director’s way of dealing with any necessary adaptations relating to the actor’s age, is practical as well as a piece of art itself. In Ibsen's original manuscript, the end of the play has the Button-moulder confronting Peer. In this production instead, the Button-moulder is played by a younger actor, and he accompanies Peer to the stage throughout, functioning as a prompter and support. This constant physical proximity and interaction between the two leave us with time for reflection upon the mysterious character of the Button-moulder. Who is he? Peer’s alter ego? A walking self-criticism sent by God?

After having travelled the world, Peer returns to Norway as an old, betrayed and disappointed man. Confronted with the Button-moulder’s order, Peer is finally forced to confront his (mis)deeds and account for his life and actions:

“Your grave is dug ready, your coffin bespoke.

The worms in your body will live at their ease; but I have orders, without delay, on Master's behalf to fetch in your soul.”

Engaged and gripped as never before, and whilst attending a funeral from a distance, Peer listens to the priest speaking at the graveside, strongly wishing that the sermon could have been said about him.

“(…) in the small circle where he saw his calling,

There he was great because he was himself.

His inborn note rang true unto the end. (…)

Yet dare I freely, firmly, speak my hope:

He scarce stands crippled now before his God!”

Peer suddenly faces all the hints he did not take, his unsung songs, his unmade works, his unwept tears, and the questions that were never asked. Faced with the universal fear; that he has been nothing, meant nothing to anyone, Peer struggles to prove that he has not been a troll, but has been himself with a soul. The Button-moulder provides him with some more time, but just until they *“meet at the next cross-roads”.*

“So unspeakably poor, then, a soul can go

Back to nothingness, into the grey of the mist.

Thou beautiful earth, be not angry with me

That I trampled thy grasses to no avail.

(…)

The spirit is niggard and nature lavish;

And dearly one pays for one’s birth with one’s life.”

Finally, marked by age, and facing death, even Peer shows signs of self-awareness, contrition and some insight into the wisdom that may accompany ageing and maturity. He asks Solveig, who has loved him and waited her whole life; *“Tell me, then where was my real self, Complete and true the Peer who bore The stamp of God upon his brow?”*[4].

And she answers;* “In my faith, in my hope, and in my love”*.

Has she saved Peer in the last minute? Ibsen left the question open.

While future productions will continue to suggest new answers, Toralv Maurstad has reached his last crossroads. Controversial at times, but foremost admired and respected. He refused to go roundabout. His soul may last and not be forgotten. However, this was how he commented on his last role: “I don’t see Peer as a liar. He has an all too lush and untamed imagination. He brags and rhymes, with charm and wit. The mistake is that he abuses his imagination to impress and to get attention—also from the ladies. It will be interesting to come to terms with such a life. I think he has to adjust to being melted down, to join the crowd among other people. As we are all perhaps expected to do [5]”.
